# Headache attributed to giant cell arteritis complicated with rheumatic polymyalgia diagnosed with F18-fluorodeoxyglucose positron emission tomography and computed tomography: a case report

**DOI:** 10.3389/fneur.2023.1241676

**Published:** 2023-09-12

**Authors:** Dong Wang, Zunjing Liu, Huailian Guo, Li Yang, Xinhua Zhang, Li Peng, Min Cheng, Hong Jiang

**Affiliations:** ^1^Department of Neurology, Peking University People's Hospital, Beijing, China; ^2^Department of Ultrasound, Peking University People's Hospital, Beijing, China

**Keywords:** headache, giant cell arteritis, rheumatic polymyalgia, PET, case report

## Abstract

Giant cell arteritis (GCA) is a kind of systemic vasculitis affecting individuals over 50 years old and is often the cause of new-onset headaches in older adults. Patients with GCA sometimes have rheumatic polymyalgia (PMR). The diagnosis of GCA generally depends on clinical manifestation, elevated erythrocyte sedimentation rate (ESR) or C-reactive protein, and positive imaging findings commonly obtained by ultrasound or temporal artery biopsy. In this study, we report a case of an 83-year-old woman with a new-onset headache and an elevated ESR. The result of the temporal artery ultrasound did not distinguish between vasculitis and atherosclerosis. The F18-fluorodeoxyglucose positron emission tomography and computed tomography (18F FDG PET-CT) were performed and suggested large vessel vasculitis with temporal artery involvement. In addition, polyarticular synovitis and bursitis were also revealed. Finally, the diagnosis of secondary headache attributed to CGA complicated with PMR was established. The patient experienced remission of symptoms after glucocorticoid therapy. PET can become a powerful tool for diagnosis and differential diagnosis when the ultrasound result is ambiguous and a biopsy is not obtained.

## Introduction

Giant cell arteritis (GCA) is a kind of systemic vasculitis involving the large and middle arteries, also known as temporal arteritis (1). The age of the population with GCA is generally over 50 years, and the annual incidence among individuals over 50 years old is ~10–20 per 100,000, which is highest in Nordic countries and rare in Asia; the average age at diagnosis is over 70 years (2). The prevalence rate of female patients is higher than that of male patients, and the ratio of male to female is 1:2.5–3 (3). GCA is closely related to rheumatic polymyalgia (PMR); 46–60% of GCA patients have comorbid PMR, and 16–21% of PMR patients have comorbid GCA (4). As the temporal artery and other cranial arteries are commonly involved, GCA is one of the causes of new-onset headache in middle-aged and elderly people (5). Although biopsy is still the golden standard of GCA, its low acceptance of invasive examination limits its uses. If the positive ultrasound result and other supportive evidence are absent, there would be a dilemma for differential diagnosis and optimal treatment. Here, we report a case of GCA complicated with PMR diagnosed by F18-fluorodeoxyglucose positron emission tomography and computed tomography (18F FDG PET-CT). Written informed consent for the personal medical information and images to be published was provided by a legally authorized representative.

## Case description

The patient, an 83-year-old woman, went to the Neurologic Clinic, at Peking University People's Hospital on May 9, 2022 because of a paroxysmal headache for 10 days. The pain was located in the right temporal region and waxed and waned, sometimes accompanied by congestion and tears in the right eye. She had no fever, no dizziness, no nausea or vomiting, no blurred vision, no jaw and tongue claudication, and no general malaise. Blood pressure was 100/70 mmHg in the left upper limb but undetectable in the right upper limb by electronic sphygmomanometer. There was no obvious tenderness in the region of the right temporal artery. The routine blood examination showed moderate anemia (hemoglobin content of 85 g/L) and an erythrocyte sedimentation rate (ESR) of 101 mm/h. Considering the possibility of a secondary headache, she was admitted to the neurology ward on May 11, 2022. The patient had a history of neurological deafness, diabetes, and hypertension, but no family history of headache or cerebrovascular disease. Two months ago, an ultrasound examination showed that sclerotic plaque was formed at bilateral subclavian arteries with the intima thickened; the distal end of the right subclavian artery was severely stenosed (about 85%), and the distal part of the left subclavian artery was moderately stenosed (60–65%). A neurological physical examination suggested a positive right Babinski and Chaddock sign, no meningeal irritation sign, and other positive signs of the nervous system.

The C-reactive protein (CRP) was elevated (39.1 mg/L). Cytokeratin 19 fragment was slightly elevated (3.32 ng/ml, reference < 3.30 ng/ml) among the tumor marker measurements. The urine and stool routine, blood biochemical analysis, antinuclear antibody, anti-ENA antibody, ANCA, Coomb's test, rheumatoid factor, immunoglobulin, galactomannan test (1,3)-β-D-glucan and galactomannan test, T-cell spot test for tuberculosis infection, PPD test, anti-double-stranded DNA antibody, serum and urine immunofixation electrophoresis, IL-2/4/6/10, IFN-γ, TNF-α, cytomegalovirus, EB virus, and adenovirus nucleic acid detection showed no markedly abnormal findings.

**Figure 1:**
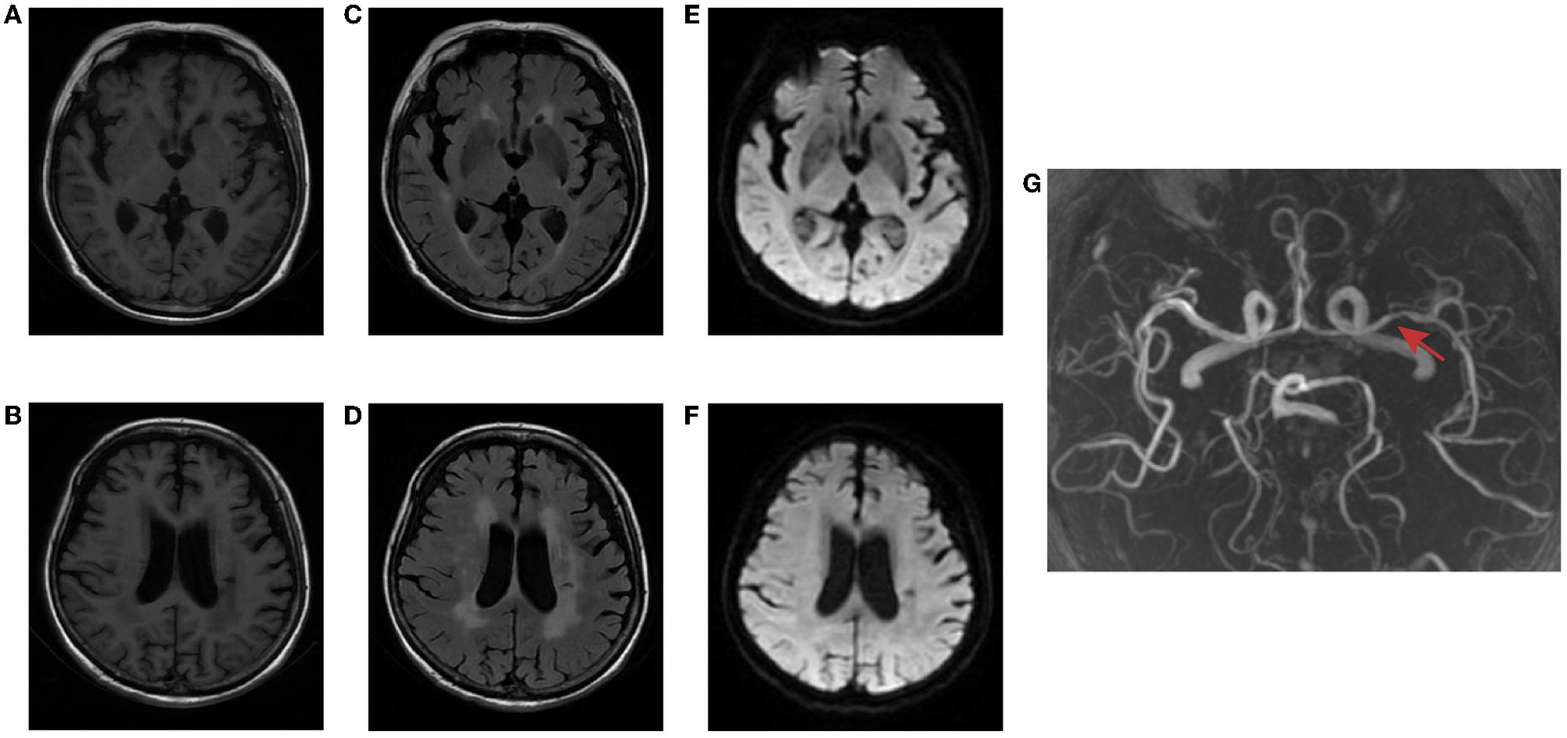
Brain MR of the patient. T1-weighted imaging **(A, B)** and FLAIR **(C, D)** showed several lacunar foci in bilateral basal ganglia and thalamus, leukoencephalopathy, and atrophy. No hyperintense on DWI **(E, F)**. MRI angiogram suggested stenosis of the left middle cerebral artery (red arrow) **(G)**.

**Figure 2:**
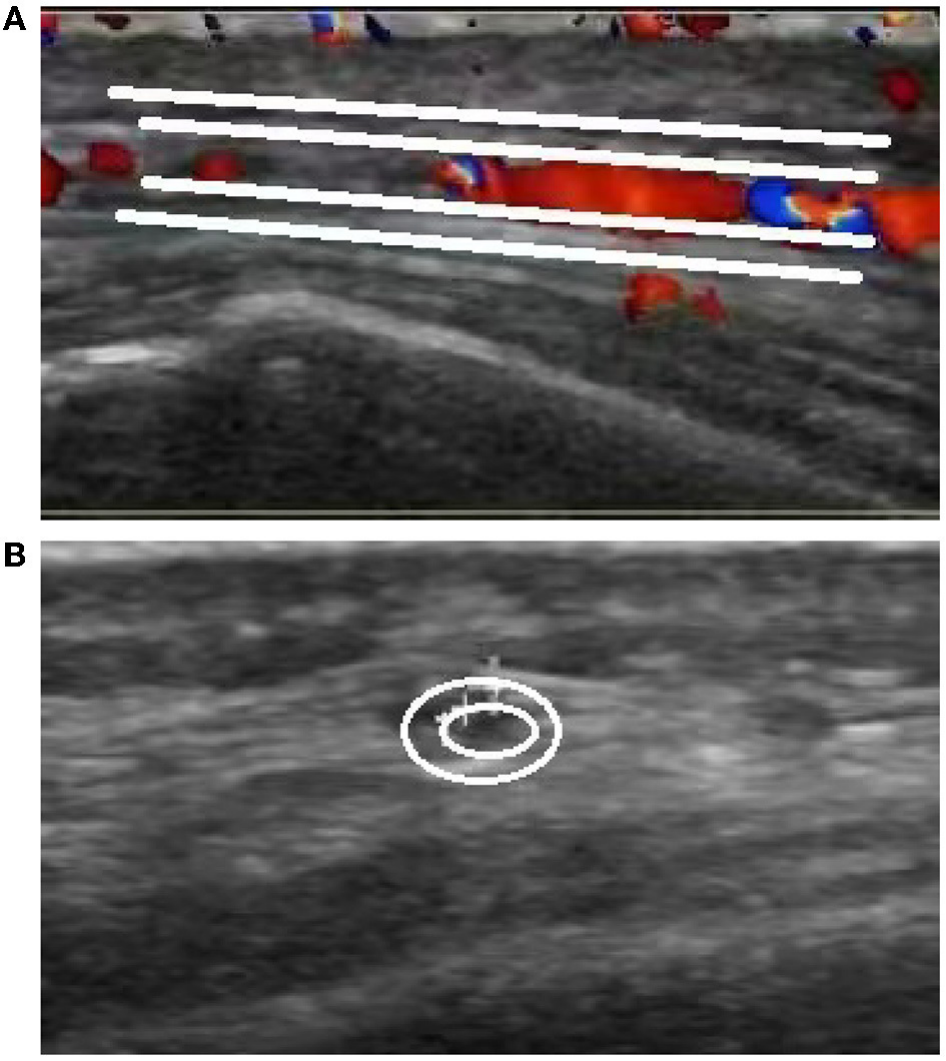
Ultrasound images of the temporal artery. Long-axis section of the temporal artery **(A)** exhibits irregular wall morphology characterized by thickening and the presence of a few punctate strong echo plaques. Short-axis section of the temporal artery **(B)** demonstrates non-uniform thickening with a non-smooth appearance.

**Figure 3:**
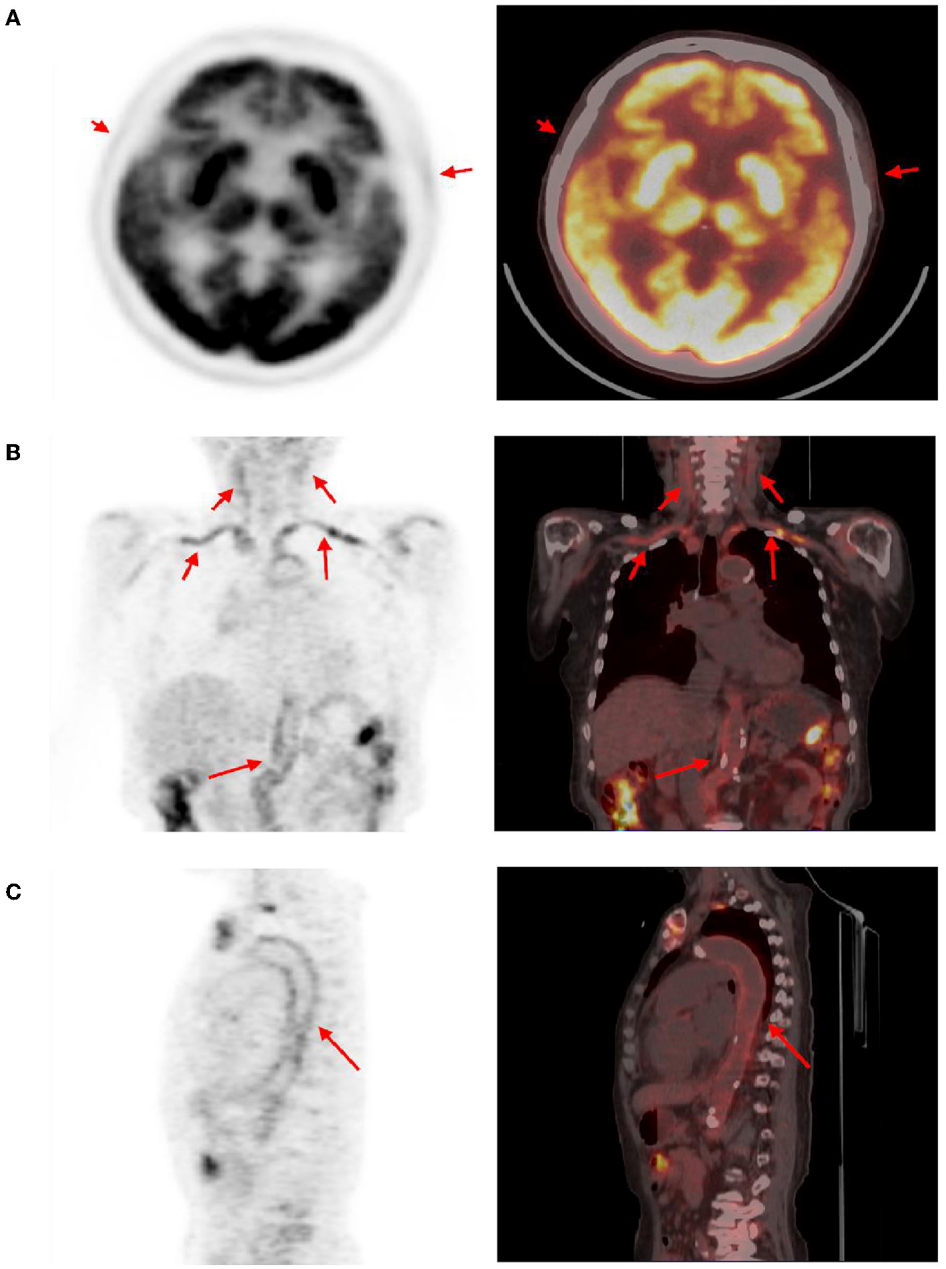
Abnormal findings of arteries in PET-CT. The temporal arteries **(A)**, subclavicular arteries, brachial trunk, axillary arteries, carotid arteries **(B)**, aortic arch, descending aorta, abdominal aorta **(C)**, and several other arteries showed an uneven increase in FDG uptake (red arrow, SUVmax distribution in 1.4–5.3), and corresponding CT showed slightly thicken vascular wall. Calcification can be seen in some artery walls.

**Figure 4:**
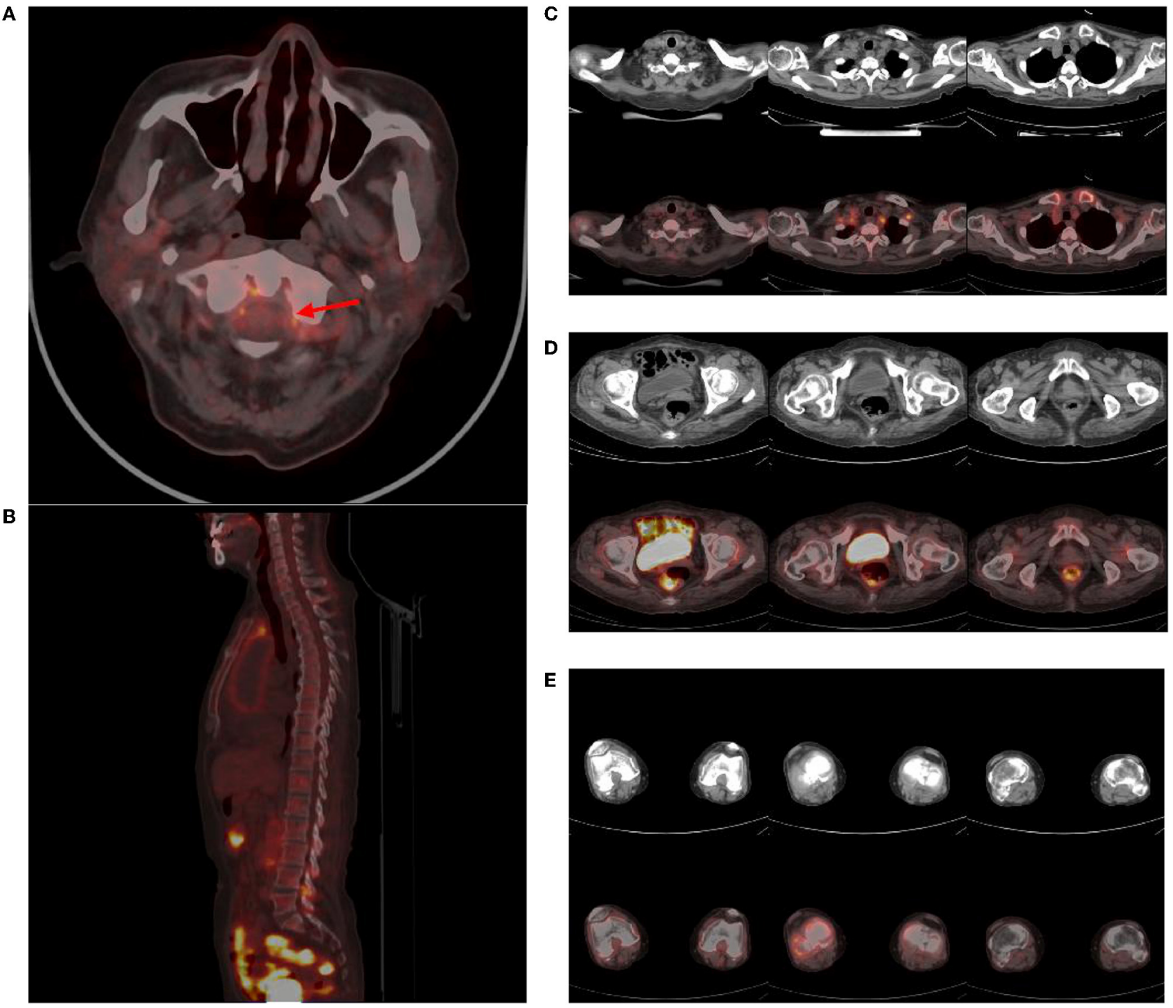
Abnormal findings of joints in PET-CT. Atlantoaxial joint (red arrow) **(A)**, lumbar spinous processes **(B)**, sternoclavicular and shoulder joints **(C)**, hip joints **(D)**, knee joints **(E)**, and several other regions showed an increase in FDG uptake (SUVmax: 4.2), and corresponding CT showed synovial thickening or bursae enlargement.

The brain MR (Figures 1A–F) showed several lacunar foci in bilateral basal ganglia and thalamus (especially on the left), leukoencephalopathy, and atrophy. An MRI angiogram (Figure 1G) suggested intracranial arteriosclerosis and mild to moderate stenosis of the M1 segment of the left middle cerebral artery. Temporal artery ultrasound (TAU) showed hypoechogenic non-smooth thickening of the bilateral superficial temporal artery wall with the presence of a few punctate strong echo plaques (Figure 2), which could not tell vasculitis from atherosclerosis clearly. After a consultation with a rheumatologist, the patient underwent an 18F FDG PET-CT examination. The results showed that FDG uptake increased unevenly in the temporal arteries, ascending aorta, aortic arch, brachiocephalic trunk, descending aorta, abdominal aorta, bilateral subclavian arteries, axillary arteries, carotid arteries, femoral arteries, popliteal arteries, and tibial arteries (SUVmax: 1.4–5.3). Vessel walls were slightly thickened, and strip calcification was seen in part of them (Figure 3). In addition, increased FDG uptake (SUVmax: 4.2) could be seen in atlantoaxial, shoulder, wrist, hip, knee, sternoclavicular joints, and lumbar spinous processes, with synovium thickened or synovial capsule enlarged (Figure 4). In the meantime, the patient presented another clinical clue that she had bilateral shoulder joint, knee joint, hip, and waist pain in the past 2 years. Finally, the diagnosis of GCA complicated with PMR was established.

She received 40 mg of intravenous methylprednisolone daily for 7 days and then 50 mg of oral prednisone daily. The headache and limb pain were significantly relieved. ESR decreased from 101 to 25 mm/h, and CRP decreased from 39.1 to 2.7 mg/L. The disease was kept under good control at follow-up.

## Discussion

This case involved an elderly woman with new-onset headache, accompanied by an elevation of ESR and CRP. Except for the positive right Babinski and Chaddock sign, which could be explained by old lesions of the brain revealed by the MRI, no other positive sign was found. The secondary headache attributed to GCA was suspected. On the basis of the GCA diagnostic criteria put forward by the American College of Rheumatology (ACR) in 1990 (6), ACR and the European League Against Rheumatism (EULAR) introduced the “halo sign” of TAU and high FDG uptake of large arteries in FDG-PET into the diagnostic criteria in 2022. After the introduction of TAU and PET, the new criteria have a sensitivity of 87% and a specificity of 95% (7). A halo sign is defined as a hypo- or iso-echogenic dark aspect of the vessel walls (8). The TAU in this case showed hypoechogenic uneven thickening of the artery wall with punctate plaques, which did not fit the typical halo sign and failed to distinguish between vasculitis and atherosclerosis (9, 10). According to EULAR's recommendations on the application of imaging in the clinical practice of large vessel vasculitis, a temporal artery biopsy (TAB) or further imaging examination was needed to confirm the diagnosis (11).

TAU is convenient and easy to perform, and it is the preferred imaging method when GCA is clinically suspected (11). According to the literature, the halo sign of TAU has a sensitivity of 77% and a specificity of 96% for the diagnosis of GCA (12). TAB is still the gold standard for diagnosing GCA (13), but biopsy is invasive, and its acceptance by patients is low. Pooled data showed that the sensitivity of TAB was similar to TAU findings, but the positive rate of biopsy showed a decreasing trend year by year (14). The patient refused an invasive examination. In recent years, studies have found that FDG-PET had high sensitivity and specificity for the diagnosis of GCA, which were 80–90% and 73–98%, respectively (15). In this case, there was no typical halo sign, which made the involvement of the temporal artery doubtful. In pathological process, functional changes often precede structural changes, and FDG-PET theoretically has a higher sensitivity to early vascular inflammation with only functional involvement. In addition, although ESR was elevated in this elderly woman, other rheumatic and immune-related indicators and pathogenic agent tests were negative. Therefore, the possibility of tumors could not be ruled out, and PET is more helpful for the differential diagnosis. Therefore, PET-CT was finally chosen.

The results of PET-CT showed that the aorta, its main branches, and even the distal arteries showed high uptake of linear FDG. Combined with other clinical features, according to the classification criteria of ACR/EULAR in 2022, the diagnosis of GCA was established (7). Meanwhile, PET-CT revealed no signs of a tumor. Although the result of TAU was ambiguous, abnormal FDG uptake of the temporal artery in PET-CT confirmed that PET could detect vessel wall inflammation before significant changes in anatomical morphology. Therefore, PET can also show the extent of disease involvement earlier and more systematically. In addition, PET can also quantitatively display the inflammation level of the blood vessel wall, and different stages of the disease can be displayed as standard uptake values (SUV) of different degrees on PET, so as to better reflect the activity of the disease (16).

Notably, polyarticular synovitis and bursitis were also detected, suggesting PMR, which was overlooked in the initial clinical inquiry related to headache. PMR lacks specific diagnostic methods, and its diagnosis depends on clinical criteria and exclusion diagnoses. PET can not only detect the presence of GCA but also reveal the abnormal FDG uptake characteristic of PMR (17). The study by Yamashita et al. suggested that when PET showed that two or more parts of the ischial tubercle, greater trochanter, and lumbar spinous process were involved, the sensitivity and specificity of diagnosing PMR were 85.7 and 88.2%, respectively. The sensitivity and specificity of the lumbar spinous process alone can also reach 78.6 and 82.4%, respectively. Other parts, such as sternoclavicular joints, shoulder joints, elbow joints, wrist joints, hip joints, knee joints, and others, also have abnormal manifestations but lack specificity (18). Other studies have also summarized the PET imaging characteristics of PMR (19, 20). Compared to ultrasound, PET imaging results are more specific (21). In this case, several regions including the spinous process of the lumbar spine were involved. Based on the patient's clinical manifestations and hematological indicators, the diagnosis of GCA complicated with PMR was established (22). The patient's response to treatment also supports the diagnosis.

The diagnosis and the treatment process, in this case, were not perfect. It can be seen from the figure that the subject's brain tissue showed high uptake of 18F-FDG, while the walls of the cerebral arteries were too thin to be detectable against the background. The patient's MRA showed stenosis of the left middle cerebral artery, and there were many old lesions in the left cerebral hemisphere. Therefore, it was not clear whether the intracranial artery was involved. Kinoshita et al. reported a case of cerebral infarction caused by GCA with middle cerebral artery involvement. After 3 months of prednisolone therapy, the patient's middle cerebral artery stenosis improved (23). Perhaps follow-up observation or other imaging examinations, such as a high-resolution MRI angiogram, can make up for the defect of PET in the evaluation of intracranial blood vessels.

PET has certain advantages in assisting the diagnosis of GCA and PMR, such as higher sensitivity and specificity, quantitative evaluation of the inflammation level, and evaluation of the disease extent involved. Of course, factors such as high costs and radiation exposure also limit its promotion. Although it cannot be used as a routine inspection, PET can become a powerful tool for diagnosis when the TAU result is negative and the TAB is not agreed upon.

## Data availability statement

The raw data supporting the conclusions of this article will be made available by the authors, without undue reservation.

## Ethics statement

The studies involving humans were approved by the Ethics Committee of Peking University People's Hospital. The studies were conducted in accordance with the local legislation and institutional requirements. The participants provided their written informed consent to participate in this study. Written informed consent was obtained from the individual(s) for the publication of any potentially identifiable images or data included in this article.

## Author contributions

HJ, ZL, and HG contributed to the conception and design of the study. DW wrote the initial manuscript. XZ, LP, and MC contributed to the clinical analysis. LY contributed to the imaging analysis. HJ contributed to the manuscript's revision. All authors read and approved the submitted version.
